# IgG Autoantibodies Against IgE from Atopic Dermatitis Can Induce the Release of Cytokines and Proinflammatory Mediators from Basophils and Mast Cells

**DOI:** 10.3389/fimmu.2022.880412

**Published:** 2022-05-31

**Authors:** Remo Poto, Isabella Quinti, Gianni Marone, Maurizio Taglialatela, Amato de Paulis, Vincenzo Casolaro, Gilda Varricchi

**Affiliations:** ^1^ Department of Translational Medical Sciences, University of Naples Federico II, Naples, Italy; ^2^ Center for Basic and Clinical Immunology Research (CISI), University of Naples Federico II, Naples, Italy; ^3^ World Allergy Organization (WAO) Center of Excellence, Naples, Italy; ^4^ Department of Molecular Medicine, Sapienza University of Rome, Rome, Italy; ^5^ Institute of Experimental Endocrinology and Oncology (IEOS), National Research Council (CNR), Naples, Italy; ^6^ Department of Neuroscience, University of Naples Federico II, Naples, Italy; ^7^ Department of Medicine, Surgery and Dentistry ‘Scuola Medica Salernitana’, University of Salerno, Baronissi, Italy

**Keywords:** allergy, anti-IgE, atopic dermatitis, basophils, IL-4, IL-13, mast cells

## Abstract

IgE-mediated release of proinflammatory mediators and cytokines from basophils and mast cells is a central event in allergic disorders. Several groups of investigators have demonstrated the presence of autoantibodies against IgE and/or FcεRI in patients with chronic spontaneous urticaria. By contrast, the prevalence and functional activity of anti-IgE autoantibodies in atopic dermatitis (AD) are largely unknown. We evaluated the ability of IgG anti-IgE from patients with AD to induce the *in vitro* IgE-dependent activation of human basophils and skin and lung mast cells. Different preparations of IgG anti-IgE purified from patients with AD and rabbit IgG anti-IgE were compared for their triggering effects on the *in vitro* release of histamine and type 2 cytokines (IL-4, IL-13) from basophils and of histamine and lipid mediators (prostaglandin D_2_ and cysteinyl leukotriene C_4_) from human skin and lung mast cells. One preparation of human IgG anti-IgE out of six patients with AD induced histamine release from basophils, skin and lung mast cells. This preparation of human IgG anti-IgE induced the secretion of cytokines and eicosanoids from basophils and mast cells, respectively. Human monoclonal IgE was a competitive antagonist of both human and rabbit IgG anti-IgE. Human anti-IgE was more potent than rabbit anti-IgE for IL-4 and IL-13 production by basophils and histamine, prostaglandin D_2_ and leukotriene C_4_ release from mast cells. Functional anti-IgE autoantibodies rarely occur in patients with AD. When present, they induce the release of proinflammatory mediators and cytokines from basophils and mast cells, thereby possibly contributing to sustained IgE-dependent inflammation in at least a subset of patients with this disorder.

## Introduction

Mast cells and basophils are important cells of the immune system (1–3) and play critical roles in several allergic (4–9) and autoimmune disorders (10–12), infections (13, 14), cardiovascular diseases (15–17), immunodeficiencies (18), and cancer (19–22). The secretion of preformed mediators (e.g. histamine) and *de novo* synthesis of lipid mediators (e.g. leukotriene C_4_, prostaglandin D_2_) and various cytokines following FcεRI cross-linkage plays key roles in diverse IgE-mediated allergic conditions, including atopic dermatitis (AD) (23), chronic spontaneous urticaria (CSU) (24, 25), asthma (5, 26, 27), allergic rhinitis (28), food allergies (29), and anaphylaxis (30–32).

Human mast cells and basophils express a complete (αβγ2), high-affinity receptor for IgE (FcεRI) (33). The interaction of IgE with its receptor is characterized by a very slow dissociation rate (K_off_ < 10^-5^/s), accounting for its uniquely high affinity, the highest reported for a human immunoglobulin (Ig) to any of its receptors (34, 35). Aggregation of FcεRI bound to IgE by multivalent antigens, anti-IgE antibodies generated in rabbit or goat (36, 37), or superantigens (38–41) leads to mast cell and basophil activation and mediator release.

Several studies have reported the presence of spontaneously occurring autoantibodies to IgE (36, 42–45), FcεRI (46–49), or both in diverse allergic (36, 42–46, 48, 50–52) and autoimmune disorders (47, 53). Most of these studies have focused on the ability of anti-IgE/FcεRI autoantibodies isolated from patients with CSU to activate peripheral blood basophils (36, 42, 46–48). However, most anti-IgE/FcεRI antibodies isolated from patients with CSU (36), asthma (50), or AD (44) are ineffective basophil secretagogues, which might explain some of the controversies in the field (50, 54). These controversial findings do not necessarily rule out the ability of some of these autoantibodies to activate human tissue mast cells. In any instance, the recent documentation of IgE autoantibodies against eosinophil peroxidase and eosinophil cationic protein in some patients with CSU and AD further reinforce the notion that shared, dysregulated immune functions may differentially contribute to the pathogenesis of these conditions (55).

Even though basophils account for approximately 1% of circulating peripheral blood leukocytes, analysis of basophil activation *in vitro* has become a mainstay of research in allergy and immunology for some compelling reasons. First, these cells can play critical roles in the activation of type 2 immune responses through the production of such Th2-like cytokines as IL-4 and IL-13 (38, 39, 56–62); second, basophils have the propensity to migrate into the sites of allergic inflammation (63–65); last, but not least, these cells are much more readily available for analysis than human tissue-resident mast cells.

The purpose of this study was four-fold. First, we examined the presence of functional IgG anti-IgE autoantibodies in patients with AD and compared their functions to rabbit IgG anti-IgE and to human polyclonal IgG. Second, we evaluated the effects of functional IgG anti-IgE on the release of Th2-like cytokines (IL-4 and IL-13) from human basophils. Third, we investigated whether human monoclonal IgE is a competitive antagonist of human and rabbit IgG anti-IgE. Finally, we examined the ability of functional human IgG anti-IgE to activate human primary skin and lung mast cells.

## Materials and Methods

### Reagents and Buffers

Bovine serum albumin, human serum albumin, piperazine-N,N’-bis (2-ethanesulfonic acid) (Pipes), L-glutamine, antibiotic-antimycotic solution (10,000 IU penicillin, 10 mg/mL streptomycin, and 25 µg/mL amphotericin B), collagenase (Worthington Biochemical Corp., Lakewood, NJ, USA), Hanks’ balanced salt solution, fetal calf serum (FCS) (Thermo-Fisher, Grand Island, NY, USA), pronase (Merck Millipore, Burlington, CA, USA), RPMI 1640 with 25 mM HEPES buffer, Eagle’s minimum essential medium (Fuji Film, Research Triangle Park, NC, USA), Percoll (Pharmacia Fine Chemicals, Uppsala, Sweden), CD117 MicroBeads (Miltenyi Biotech, Bologna, Italy), Iscove modified Dulbecco Medium (IMDM) (Fuji Film, Research Triangle Park, NC, USA), HClO_4_ (Baker Chemical Co., Deventer, Netherlands), hyaluronidase, chymopapain, elastase type I, cysteinyl leukotriene C_4_ (LTC_4_), and prostaglandin D_2_ (PGD_2_) (Sigma Chemical Co., St. Louis, MO), deoxyribonuclease I (Merck Millipore, Burlington, CA, USA), (^3^H)-LTC_4_ and (^3^H)-PGD_2_ (New England Nuclear, Boston, MA) were commercially purchased. Rabbit IgG anti-IgE antibody, produced by rabbit immunization with the Fc fragment of a human IgE myeloma (patient PS) and then absorbed with the IgE Fab as previously described (37), was kindly donated by Drs. Kimishige and Teruko Ishizaka (La Jolla Institute for Allergy and Immunology, La Jolla, CA). Rabbit anti-LTC_4_ and anti-PGD_2_ antibodies were a gift of Dr. Lawrence M. Lichtenstein (The Johns Hopkins University, Baltimore, MD). The Pipes buffer used in these experiments was a mixture of 25 mM Pipes, 110 mM NaCl, 5 mM KCl, pH 7.37, referred to as P. P2CG contains, in addition to P, 2 mM CaCl_2_ and 1 g/L dextrose (66); pH was titrated to 7.4 with NaHCO_3_.

### Atopic Dermatitis Patients

The study was approved by the Ethics Committee of the University of Naples Federico II, School of Medicine (Prot. 198/18), and informed consent was obtained from all participants prior to collection of blood according to recommendations from the Declaration of Helsinki. Serum samples from six patients with AD (aged 5 to 17 years) and six normal donors (aged 6 to 22 years) were collected and stored at -20°C. Patients with AD had similar clinical pictures, characterized by a chronic, pruritic skin eruption marked by erythema, papules, or lichenification of flexural areas of the extremities, face and neck (67). Serum samples were obtained from these patients after not taking any drug for at least one week.

### Purification of Human Monoclonal IgE

IgE myeloma protein was purified from a myeloma patient (68) by gel filtration on Sepharose G-200 followed by elution through a Sepharose CL-4B column. Analysis by sodium dodecyl-sulfate polyacrylamide gel electrophoresis of purified human monoclonal IgE proteins demonstrated a single protein with a m.w. of 180,000-200,000 D. Analysis by radioimmunoassay showed no IgG, IgM, or IgA contamination (38, 69, 70).

### Purification of Human Polyclonal IgG

Human IgG were purified by precipitation of human serum with 50% saturated NH_4_SO_4_ followed by chromatography on a DEAE-cellulose column equilibrated with 0.01 M phosphate buffer (pH 7.9), as previously described (70, 71).

### Purification of Human IgG Anti-IgE Antibody

Comparable levels of IgG anti-IgE antibodies were detected in serum samples from the six AD patients studied, which averaged 1,020 ng/ml (± 135 ng/ml), much higher than in nonatopic controls (< 50 ng/ml) (45). For affinity purification of these autoantibodies, sera (3 ml for each run) were passed through an immunosorbent Sepharose 4B column (1.2 x 5 cm) coated with IgE purified from ADZ (45). Immunosorbent-bound Ig with anti-IgE activity were eluted with glycine HCl buffer 0.2 M (pH 2.8), and the pH was rapidly readjusted by the addition of 2 M NaOH. The total content of immunoglobulins of the eluted fraction was measured by radioimmunoassay. Anti-IgE activity belonged to the IgG isotype. IgE content was less than 0.05 U/ml. The specificity and activity of IgG anti-IgE were tested as described elsewhere (45).

### Purification of Human Basophils

Basophils were purified from peripheral blood of healthy volunteers, aged 19-45 years, undergoing hemapheresis within the Immunohematology Unit at the University of Naples Federico II. Buffy coats were subjected to double-Percoll density centrifugation, which produced basophil-depleted and basophil-enriched cell suspensions (72). Basophils were purified from the basophil-enriched cell suspensions using the Basophil Isolation Kit II (Miltenyi, Biotec, Bologna, Italy). Basophils, with purity ≥ 95%, assessed by Alcian blue staining, were incubated in IMDM in the presence of activating stimuli for 4 hours (IL-4 secretion) or 18 hours (IL-13 secretion) at 37°C (38). At the end of these incubations, the cell-free supernatants were stored at -20°C for subsequent assay of IL-4 and IL-13.

### Isolation of Human Skin Mast Cells

The study was approved by the Ethics Committee of the University of Naples Federico II (Protocol: Human MC No. 7/19) and informed consent was obtained from all donors. Skin obtained from patients undergoing either mastectomy for breast cancer or elective cosmetic surgery was separated from the subcutaneous fat by blunt dissection. The tissue was finely cut into 1- to 2-mm fragments and dispersed into single-cell suspension as previously described (73). Yields with this technique ranged between 0.1 and 0.9 × 10^6^ mast cells/g of wet tissue, and purity was between 5 and 10%. Human skin mast cells (HSMCs) were further purified using a CD117 MicroBead kit cell sorting system (Miltenyi Biotec, Bologna, Italy) according to the manufacturer’s instructions. Mast cell purity using this technique ranged from 36 to 71% as assessed by Alcian blue staining.

### Isolation of Human Lung Mast Cells

Human lung mast cells (HLMCs) were purified from macroscopically normal lung tissue obtained from patients [hepatitis C virus (HCV−), hepatitis B surface Ag (HBsAg−), HIV−] affected by lung adenocarcinoma undergoing thoracic surgery (74, 75). Freshly resected lung tissue was obtained intraoperatively and was minced finely with scissors and washed extensively with Pipes buffer over Nytex cloth (120-μm pore size) (Tetko, Elmsford, NY, USA). The cells were suspended (10^6^ cells/mL) in RPMI 1640 with 5% FCS, 2 mM L-glutamine, and 1% antibiotic-antimycotic solution and incubated in 24-well plates (Falcon, Becton Dickinson, Milan, Italy). The enzymatic tissue dispersion yielded ≈5 × 10^5^ mast cells/gram of lung tissue and purity ranged from 4% to 19% (40). HLMCs were further purified using a CD117 MicroBead kit cell sorting system (Miltenyi Biotec, Bologna, Italy) according to the manufacturer’s instructions (40). Mast cell purity using this technique ranged from 58% to 82% as assessed by Alcian blue staining.

### Histamine Release From Human Basophils

Whole blood samples were processed immediately after collection to obtain leukocyte-enriched preparations (76, 77). Duplicate leukocyte aliquots were incubated (45 minutes at 37°C) in P2CG buffer with increasing concentrations of rabbit IgG anti-human IgE myeloma (patient PS; anti-IgE) or human IgG anti-IgE. Cell-free supernatants were collected and stored at −20°C for subsequent assay of histamine content using an automated fluorometric technique (78). Histamine release (HR) was expressed as percent of the total content assessed in parallel samples lysed by addition of 2% HClO_4_, minus the basal, or spontaneous release (77). Percent HR values were the means of duplicate determinations, differing by <5%. Basophil reactivity, that is, the maximal percent histamine release (HR_MAX_), and threshold sensitivity (HR_SENS_), that is, 100x the inverse of the secretagogue concentration inducing half-maximal HR (EC_50_), were calculated as described (76, 79–81).

### Histamine Release From Mast Cells

HSMCs or HLMCs (≈3 × 10^4^ mast cells per tube) were resuspended in P2CG. 0.3 mL of the cell suspensions were placed in 12 × 75 mm polyethylene tubes. 0.2 mL of each prewarmed releasing stimulus was added, and incubation was continued at 37°C for 45 min (40, 41). At the end of incubation, cells were centrifuged (1000× g, 4°C, 5 min) and supernatants were stored at –20°C for subsequent assay of histamine content. Histamine was measured in duplicate determinations with an automated fluorometric technique (78).

### IL-4 and IL-13 ELISA

IL-4 and IL-13 were assessed in duplicate samples using ELISA kits according to manifacturer’s instructions (Quantikine ELISA Kit) (R&D Systems, Minneapolis, MN, USA). The ELISA detection range was 31-2,000 pg/ml (IL-4) and 125-4,000 pg/ml (IL-13).

### Immunoassay of LTC_4_ and PGD_2_

LTC_4_ and PGD_2_ were measured in duplicate samples by radioimmunoassay (40, 82). The anti-LTC_4_ and anti-PGD_2_ antibodies are highly selective, with less than 1% cross-reactivity to other eicosanoids (82, 83).

### Statistical Analysis

Data were analyzed with the GraphPad Prism 8 software package (GraphPad Software, La Jolla, CA, USA). Values were expressed as mean ± SEM (standard error of the mean). Statistical analysis was performed using Student’s t-test or one-way analysis of variance. Values were considered significant when the probability was below the 5% confidence level (*p* < 0.05).

## Results

### Effects of Human and Rabbit IgG Anti-IgE on Histamine Release From Human Basophils

**Figure 1:**
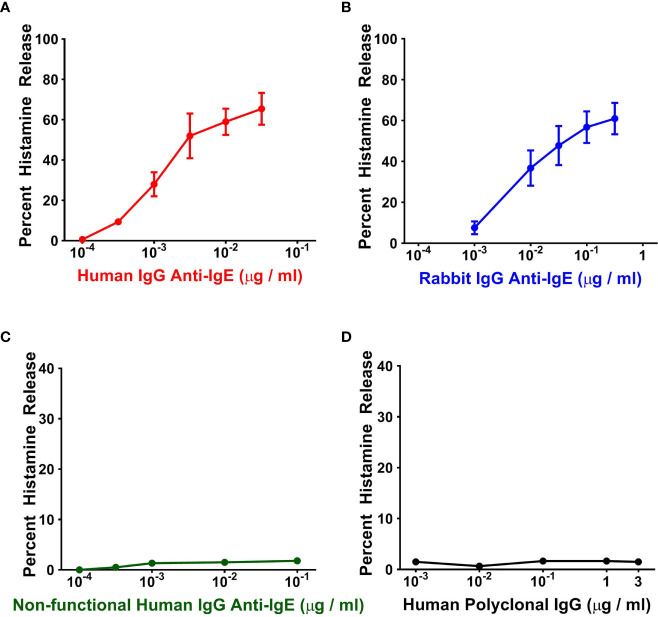
Effects of increasing concentrations of human IgG anti-IgE **(A)** and rabbit IgG anti-IgE **(B)** on HR from basophils obtained from six normal donors. Neither non-functional human IgG anti-IgE obtained from the other five atopic dermatitis donors **(C)** nor human polyclonal, pooled from six nonatopic donors, IgG induced mediator release from basophils **(D)**. Each point represents the mean ± SEM percent HR in six different preparations of basophils. Error bars are not shown when graphically too small.

In a first group of experiments, we compared the effects of increasing concentrations of human IgG anti-IgE purified from the sera of six patients with AD, rabbit IgG anti-IgE and human polyclonal IgG on HR from human basophils. **Figure 1A** shows that increasing concentrations (10^-4^ to 3 x 10^-2^ μg/ml) of human IgG anti-IgE isolated from only one out of six AD patients, as previously described (44), induced the release of substantial amounts of histamine from basophils isolated from six different normal donors. Shown for comparison is the concentration-dependent release of histamine induced by higher concentrations of rabbit IgG anti-IgE (10^-3^ to 3 x 10^-1^ μg/ml) in parallel experiments with the same basophil preparations (**Figure 1B**). Similarly, in the same experiments, non-functional human IgG anti-IgE purified from the other five AD patients did not induce HR from basophils (**Figure 1C**). In these experiments, human polyclonal IgG (10^-3^ to 3 μg/ml) purified from six healthy donors failed to induce mediator release from basophils (**Figure 1D**). Basophil reactivity, that is the maximal percent HR (HR_MAX_) in response to human IgG anti-IgE (70.0% ± 3.80%), was similar to basophil reactivity to rabbit IgG anti-IgE (65.8% ± 3.68%). By contrast, the secretagogue concentration inducing half-maximal histamine release (EC_50_) induced by the functionally active human anti-IgE preparation (2.4 x 10^-3^ ± 5 x 10^-4^ μg/ml) was significantly lower than the corresponding concentration of rabbit anti-IgE (4 x 10^-2^ ± 1 x 10^-2^ μg/ml), hence resulting in significantly higher HR_SENS_ (*p* < 0.05). These results indicate that one preparation of the human IgG anti-IgE preparations tested was active on human basophils. This preparation of human IgG anti-IgE is from now on referred to as “human anti-IgE”.

### Effects of Human and Rabbit Anti-IgE on Cytokine Production by Human Basophils

**Figure 2:**
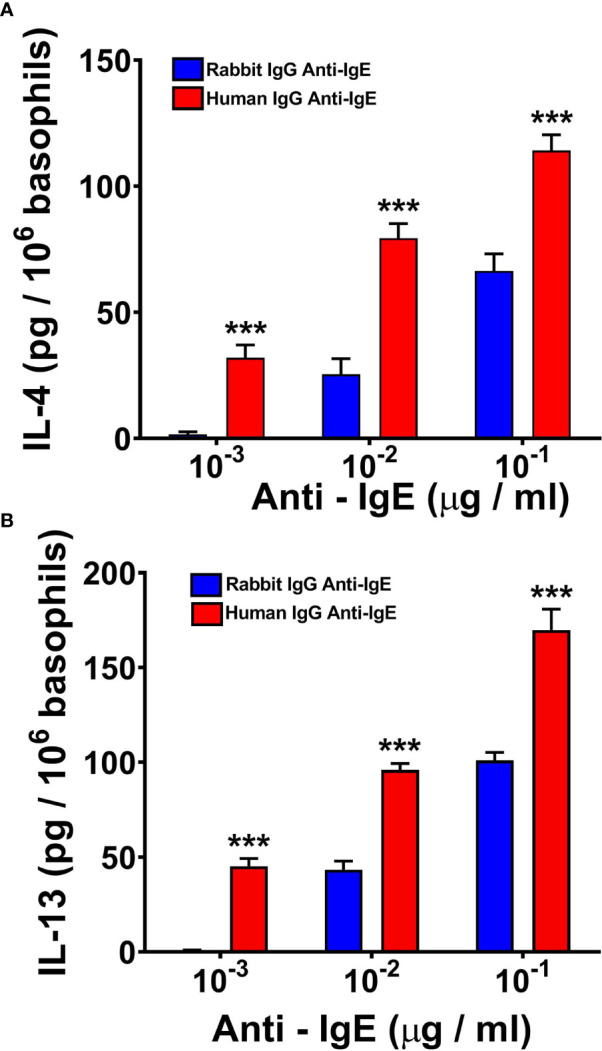
Effects of increasing concentrations of human IgG anti-IgE (red bars) and rabbit IgG anti-IgE (blue bars) on IL-4 **(A)** and IL-13 **(B)** release from human basophils obtained from five donors. Basophils were incubated with secretagogues for 4 hours (IL-4) or 18 hours (IL-13) at 37°C. Each bar represents the mean ± SEM in five parallel experiments. Error bars are not shown when graphically too small. ***p < 0.001 when compared to the corresponding value obtained with rabbit IgG anti-IgE.

IgE cross-linking induced by rabbit or goat anti-IgE (57, 58, 60, 61, 72, 84–87) or superantigens (38, 39, 59) can induce the production of IL-4 and IL-13 from human basophils. In a series of parallel experiments, we compared the effects of human and rabbit anti-IgE on the release of IL-4 and IL-13 from peripheral blood basophils purified (> 95%) from healthy donors. **Figure 2** shows the results of five independent experiments in which we examined the effects of increasing concentrations (10^-3^ to 10^-1^ μg/ml) of human and rabbit anti-IgE. In these experiments, basophils were incubated 4 hours at 37°C to evaluate IL-4 release, whereas they were incubated 18 hours at 37°C to examine IL-13 production, as previously reported (38, 39, 60, 72). Both preparations of anti-IgE induced a concentration-dependent release of IL-4 (**Figure 2A**) and IL-13 (**Figure 2B**). However, human anti-IgE, at all tested concentrations, was more effective than the corresponding concentrations of rabbit anti-IgE in inducing the release of both IL-4 and IL-13 from basophils. IgG with anti-IgE activity obtained from the other five AD patients did not cause IL-4 and IL-13 release from human basophils (data not shown). Similarly, human polyclonal IgG obtained from six normal donors did not induce cytokine release from basophils (data not shown).

### Effects of Human Monoclonal IgE on Human or Rabbit Anti-IgE-Induced Mediator Release From Human Basophils

**Figure 3:**
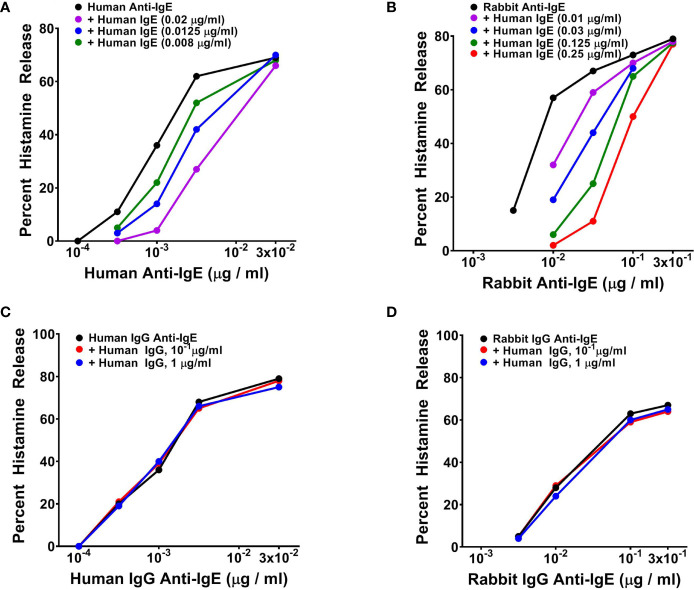
**(A)** Effects of increasing concentrations of human monoclonal IgE on human IgG anti-IgE-induced HR from human basophils. Cells were preincubated (10 minutes, 37°C) with the indicated concentrations of IgE and then challenged with the indicated concentrations of human IgG anti-IgE for an additional 30 minutes at 37°C. Each value is the mean of duplicate determinations in a typical experiment out of three similar experiments. **(B)** Effects of increasing concentrations of human monoclonal IgE on rabbit IgG anti-IgE-induced HR from human basophils. Cells were preincubated (10 minutes, 37°C) with increasing concentrations of IgE and then challenged with the indicated concentrations of rabbit IgG anti-IgE for an additional 30 minutes at 37°C. Each value is the mean of duplicate determinations in a typical experiment out of four. **(C)** Effect of increasing concentrations of human polyclonal IgG purified from a healthy donor on human IgG anti-IgE-induced HR from human basophils. Cells were preincubated (10 minutes, 37°C) with increasing concentrations of human polyclonal IgG and then challenged with the indicated concentrations of human IgG anti-IgE for an additional 30 minutes at 37°C. **(D)** Effect of increasing concentrations of human polyclonal IgG purified from a healthy donor on rabbit IgG anti-IgE-induced HR from human basophils. Cells were preincubated (10 minutes, 37°C) with increasing concentrations of human polyclonal IgG and then challenged with the indicated concentrations of rabbit IgG anti-IgE for an additional 30 minutes at 37°C. Each value is the mean of duplicate determinations in a typical experiment out of four.

The ability of human and rabbit anti-IgE to trigger basophil mediator release suggested that it might interact with basophil-bound IgE. To test this hypothesis we conducted experiments to verify whether soluble human monoclonal IgE purified from a myeloma patient (68) (70) might inhibit the mediator response to human and rabbit anti-IgE. To this end, basophils were preincubated (10 min at 37°C) with increasing concentrations of human IgE and the cells were incubated for an additional 30 min at 37°C in the presence of increasing concentrations of human or rabbit anti-IgE. **Figure 3** illustrates the results of typical experiments showing that preincubation with increasing concentrations of human monoclonal IgE concentration-dependently shifted to the right effects on basophil HR of both human (**Figure 3A**) and rabbit anti-IgE (**Figure 3B**). Preincubation (10 min at 37°C) of human basophils with tenfold higher concentrations of human polyclonal IgG did not interfere with either human (**Figure 3C**) or rabbit anti-IgE effects (**Figure 3D**). Similar results were obtained in three additional experiments. The parallel shift to the right of the HR curve caused by increasing concentrations of human monoclonal IgE on both human and rabbit anti-IgE, without changes in maximal efficacy, suggested that it might act as a competitive inhibitor.

### Effects of Human and Rabbit Anti-IgE on Histamine Release and *De Novo* Synthesis of PGD_2_ From Human Skin Mast Cells

**Figure 4:**
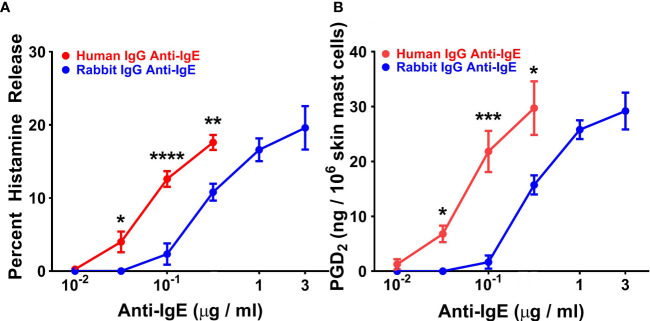
Effects of increasing concentrations of human IgG anti-IgE and rabbit IgG anti-IgE on HR **(A)** and the *de novo* synthesis of PGD_2_ **(B)** from HSMCs obtained from five different donors. HSMCs were incubated (45 min at 37°C) in the presence of the indicated concentrations of human IgG anti-IgE or rabbit IgG anti-IgE. Each point shows the mean ± SEM. **** *p* < 0.0001, *** *p* < 0.001, ** *p* < 0.01, * *p* < 0.05 when compared to the corresponding value. Error bars are not shown when graphically too small.

In five parallel experiments, we compared the activating properties of human and rabbit anti-IgE on HR (**Figure 4A**) and *de novo* synthesis of PGD_2_ by HSMCs (**Figure 4B**). The maximal percent HR caused by human anti-IgE (17.8 ± 0.91%) was similar to that induced by rabbit anti-IgE (20.2 ± 2.8%). Similarly, the maximal production of PGD_2_ induced by human anti-IgE (31.1 ± 3.7 ng/10^6^ cells) was comparable to that caused by rabbit anti-IgE (30.5 ± 2.6 ng/10^6^ cells). By contrast, the secretagogue concentration inducing half-maximal histamine release (EC_50_) for histamine release was significantly lower (5 x 10^-2^ ± 1 x 10^-2^ μg/ml) for human anti-IgE compared to rabbit anti-IgE (2.5 x 10^-1^ ± 6 x 10^-2^ μg/ml) (*p* < 0.05), indicating a comparably higher HR_SENS_. Similarly, the EC_50_ for PGD_2_ production caused by human anti-IgE (7.2 x 10^-2^ ± 2.1 x 10^-3^ μg/ml) was significantly lower than that of rabbit anti-IgE (2.9 x 10^-1^ ± 3 x 10^-2^ μg/ml) (*p* < 0.05).

### Effects of Human and Rabbit Anti-IgE on Histamine Release and *De Novo* Synthesis of Lipid Mediators From Human Lung Mast Cells

**Figure 5:**
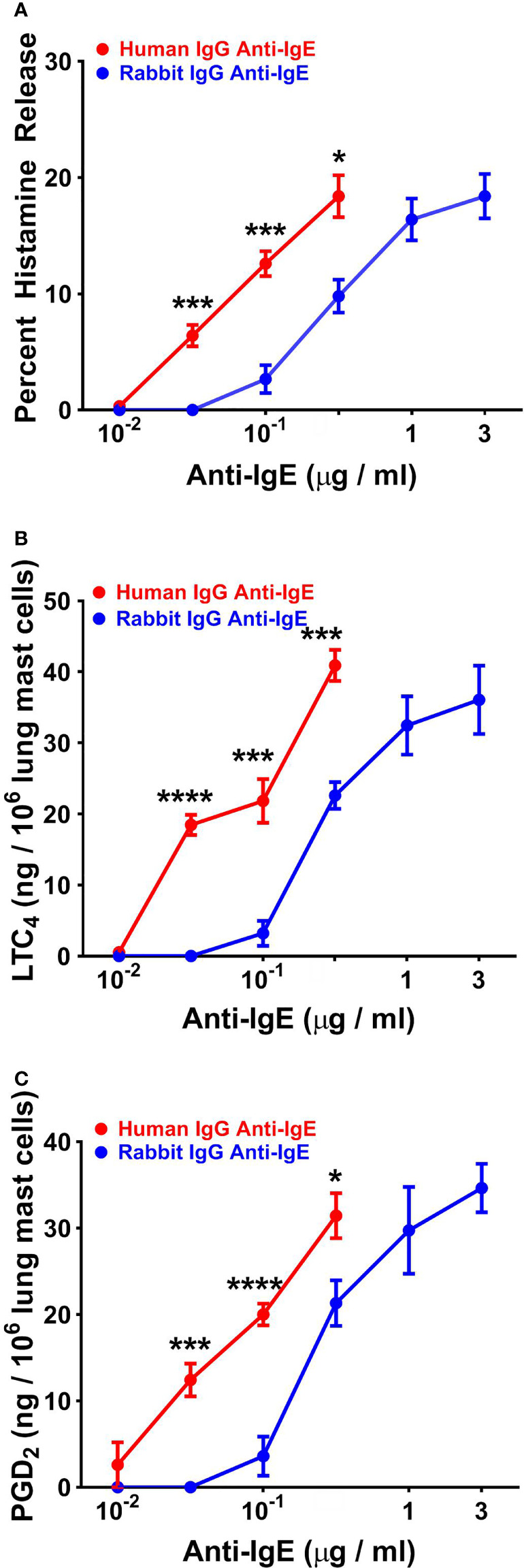
Effects of increasing concentrations of human IgG anti-IgE and rabbit IgG anti-IgE on HR **(A)** and the de novo synthesis of LTC_4_ **(B)** and PGD_2_ **(C)** from HLMCs obtained from five different donors. HLMCs were incubated (45 min at 37°C) in the presence of the indicated concentrations of human IgG anti-IgE or rabbit IgG anti-IgE. Each point shows the mean ± SEM. **** *p* < 0.0001, *** *p* < 0.001, * *p* < 0.05 when compared to the corresponding value. Error bars are not shown when graphically too small.

In five experiments, we compared the effects of increasing concentrations of human and rabbit anti-IgE on HR and *de novo* synthesis of LTC_4_ and PGD_2_ from HLMCs. Increasing concentrations (10^-2^ to 3x10^-1^ μg/ml) of human or rabbit anti-IgE (10^-1^ to 3 μg/ml) caused a concentration-dependent release of histamine from HLMCs (**Figure 5A**). The maximal percent HR in response to human anti-IgE (18.4% ± 1.8%) was similar to HLMC reactivity to rabbit anti-IgE (20.2% ± 1.2%). By contrast, the EC_50_ was significantly lower (4.6 x 10^-2^ ± 4 x 10^-3^ μg/ml) for human compared to rabbit anti-IgE (3.4 x 10^-1^ ± 8 x 10^-2^ μg/ml) (*p* < 0.01). In these experiments, we also compared the effects of human and rabbit anti-IgE on the *de novo* synthesis of LTC_4_ and PGD_2_ from HLMCs. **Figure 5B** shows that the maximal production of LTC_4_ by HLMCs exposed to human anti-IgE (40.9 ± 2.2 ng/10^6^ cells) was similar to that caused by rabbit anti-IgE (42.5 ± 2.0 ng/10^6^ cells). By contrast, the concentration of human anti-IgE inducing half-maximal LTC_4_ release was significantly lower (4.0 x 10^-2^ ± 4 x 10^-3^ μg/ml) than the EC_50_ for rabbit anti-IgE (2.5 x 10^-1^ ± 6 x 10^-2^ μg/ml) (*p* < 0.05). Similarly, HLMC reactivity to human anti-IgE (31.4 ± 2.6 ng/10^6^ cells) was similar to rabbit anti-IgE (38.9 ± 3.0 ng/10^6^ cells) with respect to PGD_2_ production (**Figure 5C**). The EC_50_ for PGD_2_ production caused by human anti-IgE (4.2 x 10^-2^ ± 1 x 10^-3^ μg/ml) was significantly lower than that of rabbit anti-IgE (2.8 x 10^-1^ ± 8 x 10^-2^ μg/ml) (*p* < 0.05).

## Discussion

Our results indicate that although autoantibodies against IgE can be found in some patients with AD, these can rarely induce the activation of human basophils and mast cells. We have detected functional IgG anti-IgE in one out of six patients with AD and characterized its ability to trigger mediator release from human basophils and mast cells. This human IgG anti-IgE is a more potent secretagogue than rabbit IgG anti-IgE, and human monoclonal IgE appears to act as a competitive antagonist of either antibody. A novel finding emerging from this study is the ability of human anti-IgE from AD to induce the release of IL-4 and IL-13 from human basophils. Another novel aspect is the observation that human anti-IgE activates not only human basophils, but also skin and lung mast cells to release histamine and arachidonic acid metabolites.

The role of naturally occurring anti-IgE/FcεRI autoantibodies in allergic and non allergic disorders is still a fascinating and unsettled issue, as recently discussed by Galli (54). Several investigators have found these autoantibodies in CSU (42, 46–49, 88–91) and in asthma (43, 50, 92). By contrast, anti-IgE autoantibodies have been inconsistently found in AD patients (43–45, 47, 52). Anti-IgE/FcεRI autoantibodies of the IgG class have been found in most of these studies (43–48, 88, 90, 91, 93, 94), while IgM (42, 49), and/or IgA autoantibodies have been only documented in rare instances (49). In most cases the autoantibodies found in patients with CSU or AD lacked the capacity to activate human basophils *in vitro* (36, 44, 47). While in some studies human IgE-specific IgG autoantibodies were able to activate human basophils (44, 47), in others they even inhibited basophil activation (36, 50).

A limitation in most of these functional studies was that they only examined the potential effects of autoantibodies to IgE or FcεRI on HR from human peripheral blood basophils (36, 42, 46–48, 88, 90, 91). The above results, while contrasting, do not necessarily rule out the hypothesis that these naturally occurring autoantibodies can activate human basophils to release cytokines (e.g., IL-4, IL-13) or tissue mast cells to produce arachidonic acid metabolites.

In this study, we found that only one preparation of human IgG anti-IgE out of six patients with AD had the ability to activate peripheral blood basophils purified from normal donors and mast cells isolated from human skin or lung tissue. Although the sample size examined in this study is too small to conclusively estimate the prevalence of functional anti-IgE autoantibodies in AD patients, these results allow to raise a few points. The apparent low prevalence of functional autoantibodies to IgE might explain, at least in part, the controversial results on the presence of functional such autoantibodies in AD patients (43–45, 47, 52). Moreover, our findings are in line with the systematic, aptly controlled observations by MacGlashan demonstrating that the autoantibodies to IgE and/or FcεRI from the vast majority of patients with CSU lacked the capacity to activate human basophil mediator release (36).

Our results provide some information on the functional potency of the IgG anti-IgE isolated from a patient with AD. Although basophil reactivity, that is the maximal HR in response to human anti-IgE, was similar to that induced by rabbit anti-IgE, the potency of human anti-IgE was significantly higher than that of rabbit anti-IgE. Similar results were obtained when comparing the reactivity and threshold sensitivity of human skin and lung mast cells to human and rabbit anti-IgE in experiments looking not only at the HR but also the *de novo* synthesis of lipid mediators (*i.e.*, PGD_2_, and LTC_4_). Collectively, these results indicate that human anti-IgE, when it is functionally present, can be significantly more potent than rabbit anti-IgE preparations commonly used in experimental or diagnostic *in vitro* protocols for IgE-dependent activation of human FcεRI^+^ cells.

We also provide some clues on the immunologic mechanism of activation of human basophils by human IgG anti-IgE. We found that preincubation of human basophils with increasing concentrations of human monoclonal IgE purified from a myeloma patient (68, 70) concentration-dependently interfered with the activating properties of both human and rabbit anti-IgE. The specificity of this response was confirmed by the observation that preincubation of basophils with tenfold higher concentrations of human polyclonal IgG did not antagonize the ability of both human and rabbit to trigger mediator release anti-IgE.

A novel finding of this study is the ability of human IgG anti-IgE to induce the release of Th2-like cytokines (e.g., IL-4, IL-13) from human basophils. The vast majority of studies exploring the functional activity of human anti-IgE and anti-FcεRI have evaluated the ability of these autoantibodies to induce HR from human basophils (36, 42, 47, 48, 50, 88, 90, 91). To the best of our knowledge, we provide the first evidence that a functional preparation of human IgG anti-IgE can also induce the release of IL-4 and IL-13 from human basophils. Also in this case, we observed that only IgG anti-IgE obtained from one out of six AD donors could cause cytokine release from basophils.

Our findings may have some translational relevance. AD is characterized by robust Th2-mediated immune responses to numerous environmental stimuli (95). The Th2 cytokines IL-4 and IL-13 are believed to play pivotal roles in the pathogenesis of AD (96, 97). Consistent with these findings, dual IL-4 and IL-13 blockade with the IL-4Rα antagonist, dupilumab showed unprecedented efficacy in adult AD patients (98, 99). Moreover, recent evidence indicates that LTC_4_ plays a role in a mouse model of AD (100). The observation that human IgG anti-IgE is a potent stimulus for the production of IL-4/IL-13 from basophils and LTC_4_ from mast cells suggests that these autoantibodies may play a role in the onset and progression of at least a subset of AD patients.

Human basophils and mast cells are key contributors to allergic disorders (1, 13, 26), including AD (67). A closer understanding of their roles in allergies has been marked by the considerable heterogeneity of these cells, whereby distinct morphologic and functional properties can not only be appreciated between mast cells and basophils (26) but also between cells located in different tissues and districts (40, 101–104). In this study, we demonstrated that human IgG anti-IgE is a potent stimulus for the production of Th2-like cytokines, hinting at a possible role in the upstream control of allergic responses, including IgE synthesis. Further, the agonist effects on prostanoids secretion from skin mast cells, mediators found at substantial levels in AD lesions (105), might have important clinical implications in AD.

In conclusion, our results extend previous findings (36, 44) indicating that only a minority of IgG anti-IgE isolated from patients with AD activates human FcεRI^+^ cells. Our data show that when functional autoantibodies to IgE are present, these can be more potent than rabbit IgG anti-IgE in inducing the release of histamine, cytokines (IL-4, IL-13) and lipid mediators (PGD_2_, and LTC_4_) from human basophils and/or mast cells. Further studies in larger cohorts of patients with different phenotypes of AD are needed to more conclusively assess the prevalence of functional autoantibodies to IgE or FcεRI and their possible contribution to disease pathogenesis and the response to current and prospective therapeutic strategies.

## Data Availability Statement

The raw data supporting the conclusions of this article will be made available by the authors, without undue reservation.

## Ethics Statement

The studies involving human participants were reviewed and approved by the Ethics Committee of the University of Naples Federico II, School of Medicine (Prot. 198/18), and informed consent was obtained from all participants prior to collection of blood according to recommendations from the Declaration of Helsinki. Written informed consent to participate in this study was provided by the participants’ legal guardian/next of kin.

## Author Contributions

RP, IQ, GM, VC, and GV designed the research. RP, IQ, AP, and VC did the experiment. RP, GM, MT, VC, and GV analyzed the data and wrote the manuscript. All authors listed have made a substantial, direct, and intellectual contribution to the work and approved it for publication.

## Funding

This work was supported in part by grants from the CISI-Lab Project (University of Naples Federico II) and TIMING Project and Campania Bioscience (Regione Campania).

## Conflict of Interest

The authors declare that the research was conducted in the absence of any commercial or financial relationships that could be construed as a potential conflict of interest.

## Publisher’s Note

All claims expressed in this article are solely those of the authors and do not necessarily represent those of their affiliated organizations, or those of the publisher, the editors and the reviewers. Any product that may be evaluated in this article, or claim that may be made by its manufacturer, is not guaranteed or endorsed by the publisher.
